# Metabolic effects of brown fat in transitioning from hyperthyroidism to euthyroidism

**DOI:** 10.1530/EJE-21-0366

**Published:** 2021-08-03

**Authors:** Lijuan Sun, Hui Jen Goh, Sanjay Verma, Priya Govindharajulu, Suresh Anand Sadananthan, Navin Michael, Yaligar Jadegoud, Christiani Jeyakumar Henry, S Sendhil Velan, Pei Shan Yeo, Yingshan Lee, Brenda Su Ping Lim, Huiling Liew, Chee Kian Chew, Timothy Peng Lim Quek, Shaikh A K K Abdul Shakoor, Wai Han Hoi, Siew Pang Chan, Daniel Ek Chew, Rinkoo Dalan, Melvin Khee Shing Leow

**Affiliations:** 1 Singapore Institute for Clinical Sciences, Agency for Science, Technology and Research (A*STAR), Singapore; 2 Institute of Bioengineering and Bioimaging, Agency for Science, Technology and Research (A*STAR), Singapore; 3 Singapore Institute of Food and Biotechnology Innovation, Agency for Science, Technology and Research (A*STAR), Singapore; 4 Department of Biochemistry, Yong Loo Lin School of Medicine; 5 Departments of Physiology & Medicine, National University of Singapore (NUS), Singapore; 6 Lee Kong Chian School of Medicine, Nanyang Technological University (NTU), Singapore; 7 Department of Endocrinology, Tan Tock Seng Hospital (TTSH), Singapore; 8 Yong Loo Lin School of Medicine, National University of Singapore, Singapore; 9 Cardiovascular and Metabolic Disorders Program, Duke-NUS Medical School, Singapore

## Abstract

**Objective:**

Brown adipose tissue (BAT) controls metabolic rate through thermogenesis. As its regulatory factors during the transition from hyperthyroidism to euthyroidism are not well established, our study investigated the relationships between supraclavicular brown adipose tissue (sBAT) activity and physiological/metabolic changes with changes in thyroid status.

**Design:**

Participants with newly diagnosed Graves’ disease were recruited. A thionamide antithyroid drug (ATD) such as carbimazole (CMZ) or thiamazole (TMZ) was prescribed in every case. All underwent energy expenditure (EE) measurement and supraclavicular infrared thermography (IRT) within a chamber calorimeter, as well as ^18^F-fluorodeoxyglucose (^18^F-FDG) positron-emission tomography/magnetic resonance (PET/MR) imaging scanning, with clinical and biochemical parameters measured during hyperthyroidism and repeated in early euthyroidism. PET sBAT mean/maximum standardized uptake value (SUV mean/max), MR supraclavicular fat fraction (sFF) and mean temperature (Tscv) quantified sBAT activity.

**Results:**

Twenty-one (16 female/5 male) participants aged 39.5 ± 2.5 years completed the study. The average duration to attain euthyroidism was 28.6 ± 2.3 weeks. Eight participants were BAT-positive while 13 were BAT-negative. sFF increased with euthyroidism (72.3 ± 1.4% to 76.8 ± 1.4%; *P* < 0.01), but no changes were observed in PET SUV mean and Tscv. Significant changes in serum-free triiodothyronine (FT3) levels were related to BAT status (interaction *P* value = 0.04). FT3 concentration at hyperthyroid state was positively associated with sBAT PET SUV mean (*r* = 0.58, *P* = 0.01) and resting metabolic rate (RMR) (*P* < 0.01).

**Conclusion:**

Hyperthyroidism does not consistently lead to a detectable increase in BAT activity. FT3 reduction during the transition to euthyroidism correlated with BAT activity.

## Introduction

Functional brown adipose tissue (BAT) has been demonstrated in human adults (1, 2, 3). BAT dissipates excess stored energy in the form of heat via non-shivering thermogenesis (NST) and is involved in the regulation of energy expenditure (EE). However, BAT's functional significance in human adults remains unclear. Both experimental and clinical studies have found an inverse relationship between BAT activity and obesity (4, 5). Activated BAT oxidizes lipids for fuel preferentially and it also utilizes glucose as a metabolic substrate. BAT might thus be implicated as a therapeutical target to combat obesity for its lipid and glucose-lowering effects (6, 7). The thermogenic function of BAT makes it a potential target for tackling obesity (8). Indeed, because of its very high metabolic activity, even a small quantity of functional BAT can have a profound metabolic impact (2).

Two chief forms of thyroid hormones, thyroxine (T4) and its active metabolite 3,5,3ʹ-triiodothyronine (T3), regulate metabolic processes that control energy utilization. Iodothyronine deiodinase types 1 and 2 (DIO1 and DIO_2_) catalyze the conversion of T4 to its active form T3 (9) that is essential for BAT differentiation and activity (10). The mechanisms of T3 and T4 in the regulation of BAT are well studied in rodent models. The T3 receptor isoform in BAT, TRβ, is required for UCP1 induction and is essential for BAT function (11). Systemic hyperthyroidism or administration of T3 in rats activated the sympathetic nervous system and induced BAT (12). Hyperthyroid mice have more BAT, higher fatty acid oxidation, and glucose uptake (13). On the contrary, the thermogenic activity of BAT is greatly reduced in the absence of T3 (14). It has been well known that T3 also contributes to BAT induction via mitochondrial biogenesis and MTOR-mediated mitophagy pathway (15). T3 is thus critical to BAT activation and white adipose tissue (WAT) browning (16, 17, 18). Rodent studies have proven that thyroid hormones accentuate glucose uptake in BAT, with hyperthyroid mice demonstrating heightened ^18^F-fluorodeoxyglucose (^18^F-FDG) uptake in BAT while the converse is true among hypothyroid mice (13).

Although thyroid hormones influencing BAT function in lower mammals have been extensively studied, their effects on BAT activity in humans are not clear. A few published human studies have shown contradictory results regarding how thyroid hormone status influences BAT function. Skarulis *et al.* (19) reported levothyroxine replacement in hypothyroid patients with increased ^18^F-FDG uptake of BAT in the lower neck and suprascapular regions (19). Lahesmaa *et al.* (20) also reported that hyperthyroidism patients have a three-fold higher BAT glucose uptake compared with the euthyroid state. However, Zhang *et al.* (21) did not detect active BAT in the hyperthyroid patients which suggested that high levels of circulating thyroid hormones may not consistently induce BAT activity. The reasons for these discrepancies are unclear. Differences in protocols used for PET-CT scanning and the ethnicity of the research participants may have led to these inconsistent results as reported in the existing literature. To gain further insight regarding the effect of thyroid hormone status on BAT in adult humans and whether BAT activity was altered during the transition from hyperthyroidism to euthyroidism, our study examined the activity of BAT determined by ^18^F-FDG PET, MR, and IRT, together with physiological and metabolic changes that occur during the transition from hyperthyroidism to euthyroidism following medical treatment in South East Asian patients with Graves’ disease.

## Subjects and methods

### Study participants

Participants between 21 and 65 years of age with newly diagnosed Graves’ disease attending the endocrine outpatient clinics of a local general hospital were recruited between October 2017 and June 2019. Graves’s disease was diagnosed based on laboratory and clinical evidence of primary hyperthyroidism and a positive serum TSH receptor autoantibody. They were enrolled if antithyroid drugs (ATD)(carbimazole/thiamazole) were not initiated for more than a month and if their latest thyroid function test (TFT) still reflected frank biochemical hyperthyroidism. Patients who were pregnant or contemplating pregnancy, allergic to carbimazole (CMZ), thiamazole (TMZ), taking medication which may affect body composition (e.g. steroids) or BAT (e.g. beta-blockers), or have a history of claustrophobia which hindered MRI scanning were excluded.

The study was conducted according to the ethical guidelines of the Declaration of Helsinki, and all procedures were approved by the Domain-Specific Review Board of National Healthcare Group, Singapore (Ethics code C/2015/00718 – TRIBUTE Study) and registered with ClinicalTrials.gov (NCT03064542). Written informed consent was obtained from all subjects before participation.

### Study protocol

**Figure 1:**
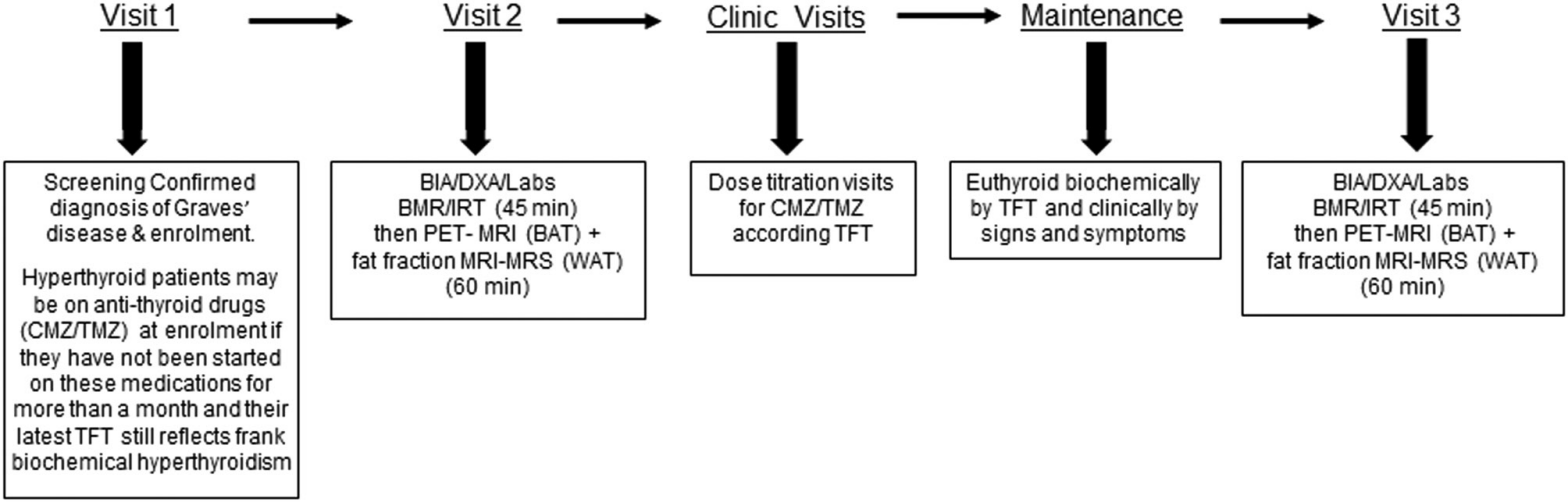
Study protocol. CMZ, carbimazole; TMZ, thiamazole; TFT, thyroid function test; Hx, PE, BIA, bioelectrical impedance analysis; DXA, dual-energy X-ray absorptiometry; BMR, basal metabolic rate; IRT, infrared thermography; PET, positron-emission tomography; MRS, magnetic resonance spectroscopy.

The study protocol is shown in Fig. 1. The patients participated in a screening visit to evaluate eligibility. Once the participants had consented to participate, they received standard ATD therapy using a decremental dosing regimen with CMZ or TMZ. The patients then were scheduled for Visit 2 for baseline research measurements while they were still hyperthyroid.

On study visit 2, patients went to the Clinical Nutrition Research Center (CNRC) in the morning at 8:30 h after an overnight fast of 8–10 h. All the patients underwent anthropometry (i.e. measures of body weight, height, waist, and hip circumference) and body composition evaluation using dual-energy X-ray absorptiometry (DXA) which measures fat, lean, and bone mass. After finishing the measurements, they underwent metabolic rate measurement in a whole-body calorimeter coupled with infrared thermography (IRT) using a thermal camera mounted on a tripod stand focusing on patients’ neck and the area above the collar bone in a whole-body room calorimeter for the next 45 min.

Then, they proceeded to the Clinical Imaging Research Center (CIRC). The patients were given an i.v. injection of radiolabeled glucose (^18^F-FDG) through the i.v. cannula and followed by fusion PET-fat fraction MRI scanning for BAT and abdominal white fat for the next 1 h.

After completion of visit 2 procedures, the patients were required to continue with follow-up at the endocrinology clinic for control of their hyperthyroidism. Clinic follow-up reviews occurred at 6–8 weekly intervals as per standard medical practice and ATD doses titrated against thyroid function tests and symptoms till euthyroidism was achieved. This took up to about 6 months. Subsequently, the patients continued with study visit 3 in exactly the same fashion as study visit 2. The measurements performed during visit 3 included anthropometry, body composition using DXA, metabolic rate, temperature in the patients’ neck area, BAT, and abdominal white fat via PET/MR scan.

### Clinical measurements

Fat mass, bone mass, and lean mass were measured by dual-energy X-ray absorptiometry (DXA) (QDR 4500A, fan-beam densitometer, software version 8.21; Hologic, Waltham, USA). Body composition measurement was performed in all study participants at baseline (during hyperthyroidism) and after achieving euthyroidism.

Resting energy expenditure (REE) was assessed through gaseous exchanges using a dual-chamber whole-body calorimeter (WBC) facility located at the Clinical Nutrition Research Center (CNRC). The WBC chambers are open-circuit air-tight indirect calorimeters and provide EE, fat oxidation (FOX), and respiratory quotient (RQ) readouts based on oxygen consumption and carbon dioxide production. The WBC has been described in detail in our previous published paper (22). REE, RQ, and FOX were measured before and after an intervention. Thermal imaging was performed in the same room as the WBC EE measurements, at a constant ambient temperature of 24°C. Anterior supraclavicular temperature is defined as Tscv. The details were reported in our previous publications (23, 24). Fasting glucose, insulin, total cholesterol, high-density lipoprotein (HDL) cholesterol, low-density lipoprotein (LDL) cholesterol, triglyceride, free T4 (FT4), free T3 (FT3), TSH, renal, and liver function parameters were determined by the National University Hospital referral laboratory.

All PET and MR scans were performed by using a hybrid PET-MR system (Biograph mMR, Siemens Healthcare) for 60 min. Intravenous injection of ^18^F-FDG (3 mCi) was administered, 40 min before imaging. High resolution 3D T1-weighted anatomical images were acquired using repetition time (TR) = 5 ms, echo time (TE) = 2.46 ms, field of view (FOV) = 384 × 288 mm^2^; matrix size =384 × 288, slice thickness = 1 mm. A 3D multi-point gradient echo (6 echoes) was used for the fat-water imaging with TR, 15 ms; TE’s, 1.1–8.61 ms (□TE = ~1.5 ms); flip angle (FA), 3°; FOV, 384 × 288 mm^2^; matrix size 192 × 144; readout mode, unipolar; 96 slices with 2 mm thickness. A graph cut algorithm was utilized for fat-water separation using the ISMRM fat-water Toolbox (25, 26). sBAT depots were manually segmented based on anatomical information in multiple slice images of registered MR and PET images using ITK-SNAP under the close guidance of an experienced clinical radiologist (27). A lower threshold of 40% of sFF values was used to exclude the muscle and bone marrow prior to computation of mean MR FF, PET SUV mean, and PET SUV max in the segmented sBAT region (24, 28). The cut-off value of sBAT PET SUV max for categorizing subjects into BAT-positive and BAT-negative groups was 1.5 (29, 30).

MR images of abdominal fat were acquired using 2-point Dixon on the hybrid MR-PET system (Biograph mMR, Siemens). A fully automated graph theoretic segmentation algorithm was used to separate and quantify the s.c.AT and visceral adipose tissue (VAT) depots and level sets-based algorithm separated the deep (DSAT) and superficial (SSAT) s.c. adipose tissue depots (23, 31).

### Statistical analysis

Differences between BAT-positive and BAT-negative were assessed using the Student’s *t*-test. Paired* t-*tests were used to compare the differences in the parameters between baseline and after an intervention. The linear mixed-effects model was used to evaluate the main effects of patient status effects (hyperthyroidism and euthyroidism), BAT status effects (BAT-positive and BAT-negative) as well as their interactions. Spearman correlations were used to assess relationships between variables after adjusting for body weight. Data are presented as means ± s.e.m., unless otherwise stated. A *P*-value ≤ 0.05 was considered statistically significant. Statistical analysis was performed by using SPSS software version 23 (IBM SPSS Inc.).

## Results

### Clinical measurements

Thirty participants with recently diagnosed Graves’ disease were recruited into the study. Twenty-one participants completed the study. The average duration of treatment required to reach euthyroidism was 28.6 ± 2.3 weeks. Clinical, biochemistry and imaging characteristics of total participants during hyperthyroidism and early euthyroidism states were summarized in Table 1. There was a significant increase in body weight from hyperthyroidism to euthyroidism (*P* < 0.01), as well as lean mass (*P* < 0.01), and fat mass (*P* = 0.04). However, there was no significant change in percentage body fat (*P* = 0.71).

**Table 1:** Characteristics of total participants during hyperthyroidism before treatments and early euthyroidism state. *n* = 21. Data are presented as mean ± S.E.M. BMI was calculated as body weight (kg) divided by the square of height (m). Homeostasis model assessment of insulin resistance was calculated as fasting glucose × fasting insulin divided by 22.5.

	Hyperthyroidism	Euthyroidism	*P*-values
BMI (kg/m^2^)	21.7 ± 0.9	22.9 ± 1.1	<0.001
Body weight (kg)	55.6 ± 2.6	58.5 ± 2.9	<0.001
Body fat (%)	34.7 ± 1.8	34.5 ± 1.8	0.71
Fat mass (kg)	19.5 ± 1.6	20.3 ± 1.7	0.04
Lean mass (kg)	33.8 ± 1.6	35.7 ± 1.8	<0.001
RMR (kcal/day)	1718.6 ± 87.7	1430.2 ± 65.2	<0.001
RQ	0.82 ± 0.01	0.84 ± 0.01	0.001
FOX	0.08 ± 0.01	0.06 ± 0.004	<0.001
Fasting glucose (mmol/L)	4.4 ± 0.1	4.5 ± 0.1	0.71
Fasting insulin (µU/mL)	5.4 ± 0.7	6.1 ± 0.8	0.33
HOMA-IR	1.1 ± 0.1	1.2 ± 0.2	0.39
Total cholesterol(mmol/L)	4.6 ± 0.2	5.6 ± 0.3	<0.001
LDL (mmol/L)	2.6 ± 0.2	3.3 ± 0.2	<0.001
HDL (mmol/L)	1.4 ± 0.1	1.7 ± 0.1	<0.001
Triglyceride (mmol/L)	1.4 ± 0.2	1.2 ± 0.2	0.06
NEFA(mmol/L)	0.7 ± 0.1	0.6 ± 0.1	0.26
SAT (cm^3^)	54.7 ± 5.9	53.9 ± 5.5	0.21
VAT (cm^3^)	22.9 ± 2.9	22.6 ± 3.0	0.68
DSAT (cm^3^)	31.8 ± 4.2	30.2 ± 3.9	0.9
SSAT (cm^3^)	22.9 ± 1.8	23.7 ± 1.7	<0.001
FT3 (pmol/L)	10.5 ± 0.9	5.5 ± 0.3	<0.001
FT4 (pmol/L)	29.9 ± 3.0	12.3 ± 1.3	<0.001
Creatinine (µmol/L)	51.9 ± 3.3	64.3 ± 4.6	<0.001
Albumin (g/L)	40.2 ± 0.6	43.4 ± 0.5	<0.001
Bilirubin conjugated(µmol/L)	3.1 ± 0.4	2.5 ± 0.3	0.007
AST (U/L)	28.6 ± 2.4	21.0 ± 1.0	0.003
ALT (U/L)	41.0 ± 6.0	19.1 ± 2.0	0.001

*P* values represent the Student’s *t*-test between hyperthyroidism and early euthyroidism, phases.

ALT, alanine aminotransferase; AST, aspartate aminotransferase; DSAT, deep SAT; FOX, fat oxidation rate; FT3, free triiodothyronine; FT4, free thyroxine; HDL, high-density lipoprotein; HOMA-IR, homeostasis model assessment of insulin resistance; LDL, low-density lipoprotein; NEFA, non-esterified fatty acids; RMR, resting metabolic rate; RQ, respiratory quotient; s.c.AT, s.c. adipose tissue; SSAT, superficial SAT; VAT, visceral adipose tissue.

The baseline RMR of the participants was significantly higher in the hyperthyroid state compared with the euthyroid state (*P* < 0.001). RQ increased significantly and FOX decreased significantly from the hyperthyroid to the euthyroid state. There were no significant changes in serum glucose, insulin levels, or HOMA-IR. Serum total cholesterol, LDL-cholesterol, HDL-cholesterol increased significantly from the hyperthyroid to the euthyroid state (*P* < 0.001). No significant changes were found in triglyceride and NEFA levels.

There was a significant increase in SSAT, but no significant changes in SAT, VAT, and DSAT from hyperthyroidism to euthyroidism. There were significant increases in creatinine and albumin level (*P* < 0.001), significant decreases in bilirubin conjugated, AST, and ALT concentrations from hyperthyroidism to euthyroidism.

### sBAT activity assessed using PET, MR, and IRT imaging

sBAT activity measured via PET, MR, and IRT in all the participants were shown in Table 2. There were no significant changes in BAT SUV mean, BAT SUV max, and BAT Tscv mean from hyperthyroidism to euthyroidism at room temperature. There was a significant increase in BAT sFF from the hyperthyroid to the euthyroid state (72.3 ± 1.4; 76.8 ± 1.4, respectively; *P* < 0.01).

**Table 2:** Changes in PET BAT SUV, MR FF, and IRT Tscv mean measured in the hyperthyroid and euthyroid state in 21 patients. *n* = 21. Data are presented as mean ± s.e.m.

	Mean ± s.e.m.	Range	*P*-values
BAT SUV mean (g/mL)			
Hyperthyroid	0.9 ± 0.1	0.4–1.9	
Euthyroid	0.8 ± 0.1	0.1–1.5	0.93
Change	–0.01 ± 0.1	–1.02–0.07	
BAT SUV max (g/mL)			
Hyperthyroid	1.7 ± 0.2	0.8–4.3	
Euthyroid	1.9 ± 0.2	0.3–4.4	0.52
Change	0.2 ± 0.3	–2.8–2.8	
BAT FF (%)			
Hyperthyroid	72.3 ± 1.4	60.6–83.2	
Euthyroid	76.8 ± 1.4	63.9–86.5	<0.01
Change	4.4 ± 0.7	1.1–10.4	
BAT Tscv mean (°C)			
Hyperthyroid	35.7 ± 0.1	35–36.3	
Euthyroid	35.6 ± 0.1	34.5–36.7	0.69
Change	–0.04 ± 0.1	–0.4–1.1	

*P* values represent the Student’s *t*-test between hyperthyroidism and early euthyroidism phases.

BAT, brown adipose tissue; FF, fat fraction; IRT, infrared thermography; MR, magnetic resonance; SUV, standardized uptake value; Tscv, anterior supraclavicular temperature.

### Imaging and clinical parameters between BAT-positive and BAT-negative participants

**Figure 2:**
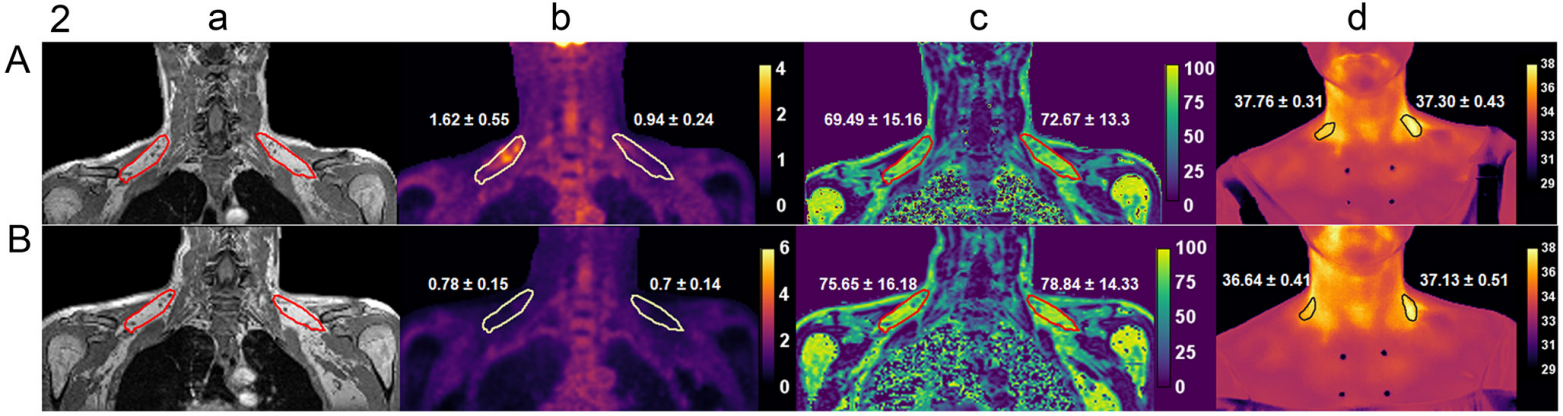
Representative images highlighting supraclavicular brown adipose tissue (sBAT) from a BAT-positive subject (A) and a BAT-negative subject (B) at hyperthyroidism (top) and euthyroidism stage (bottom). (a) T1-weighted anatomical MR image sBAT, (b) ^18^F-FDG PET (c) the fat fraction (FF) map. (d) IRT map with color bar showing the temperature in °C. The PET, FF, and IRT maps in which color scale shows values of standardized uptake values (SUV), FF (in %), and Tscv, respectively. The regions of interest (ROI) drawn around sBAT on the anatomical MR images, PET, FF, and IRT maps showing the location of sBAT. The values (mean ± s.d.) of SUV, FF, and Tscv are shown of the corresponding region of interest.

Representative images of PET, MR, and IRT from a BAT-positive and a BAT-negative participant in hyperthyroid and euthyroid state were shown in Fig. 2.

We separated the 21 subjects based on BAT status (BAT-positive and BAT-negative subjects) to examine the patients’ status and BAT status effects (Table 3). Not all the participants had detectable sBAT at room temperature in the hyperthyroid state. Participants were divided into two groups: those with detectable ^18^F-FDG uptake with a PET/MR SUV max ≥ 1.5 (BAT-positive; *n* = 8) and those with no detectable ^18^F-FDG uptake (BAT-negative; *n* = 13) according to ^18^F-FDG uptake and co-registered with MR FF as per our previous protocol (32). For PET SUV max, there was a significant association with BAT status (*P* = 0.008) but not patients’ thyroid status. Tscv mean was affected only by BAT status and not by thyroid hormone level. BAT-positive participants had a higher Tscv mean compared with BAT-negative participants. MR FF was affected by BAT status (*P* = 0.014) and also by patients’ thyroid status (*P* < 0.001). For RMR, this was significantly associated with patients’ thyroid status (*P* < 0.001) but no significant association was found with BAT status or their interaction. For total cholesterol, there was a significant association with patients’ thyroid status (*P* < 0.001) and BAT status (*P* = 0.044), but not for their interaction. For LDL-cholesterol and triglyceride, there were significant patients status effects and marginally significant BAT status effects (*P* = 0.08 and *P* = 0.06, respectively). For FT3, there were significant effects for patients status (*P* < 0.001), BAT status (*P* = 0.04), and interaction (*P* = 0.04). FT3 concentration decreased from hyperthyroid to euthyroid state which in turn were affected by BAT status. For FT4, correlation with the patients’ status (*P* < 0.001) and their interaction (*P* = 0.015) were highly significant, whereas a marginally significant association was found with BAT status (*P* = 0.083).

**Table 3:** Imaging and clinical parameters of BAT positive and BAT negative patients at hyperthyroid and euthyroid state. *n* = 21. BAT-positive *n*  =8, BAT-negative *n*  =13. Data are presented as mean ± s.e.m. The patients’ status effects, BAT status effects, and interactions between patients’ status and BAT status were tested using linear mixed-effects model.

	Hyperthyroid		Euthyroid		*P* values		
	BAT-positive	BAT-negative	BAT-positive	BAT-negative	Patients status effects	BAT status effects	Interaction
SUVmax (g/mL)	2.4 ± 0.3	1.3 ± 0.1	2.1 ± 0.4	1.8 ± 0.3	0.708	**0.008**	0.283
sFF (%)	68.1 ± 1.5	74.9 ± 1.8	72.7 ± 1.1	79.3 ± 1.9	**<0.001**	**0.014**	0.897
Tscv mean (°C)	35.9 ± 0.1	35.5 ± 0.1	36.0 ± 0.1	35.4 ± 0.1	0.907	**0.006**	0.265
RMR (kcal/day)	1839 ± 171	1645 ± 95	1494 ± 151	1391 ± 55	**<0.001**	0.340	0.353
Total cholesterol (mmol/L)	4.0 ± 0.3	5.0 ± 0.3	4.9 ± 0.4	6.0 ± 0.4	**<0.001**	**0.044**	0.838
LDL (mmol/L)	2.1 ± 0.2	2.9 ± 0.3	2.9 ± 0.3	3.6 ±0.3	**0.001**	0.077	0.845
Triglyceride (mmol/L)	1.0 ± 0.1	1.6 ± 0.3	0.7 ± 0.1	1.5 ± 0.3	**0.024**	0.06	0.094
FT3 (pmol/L)	13.0 ± 1.7	8.9 ± 0.9	5.9 ± 0.6	5.2 ± 0.3	**<0.001**	**0.037**	**0.04**
FT4 (pmol/L)	37.9 ± 4.6	25.0 ± 3.3	12.8 ± 1.7	11.9 ± 1.9	**<0.001**	0.083	**0.015**

Statistically significant values are in bold.

### Relationship of FT3 and FT4 and BAT-related markers

There was a positive association between FT3 level in hyperthyroid state and PET SUV mean (*r* = 0.58, *P* = 0.01) after adjustingt for weight, but not in the euthyroid state (Table 4). FT3 was positively associated with Tscv mean in euthyroid state (*r* = 0.55, *P* = 0.01). RMR was positively associated with FT3 both in hyperthyroid state (*r* = 0.69, *P* = 0.001) and euthyroid state (*r* = 0.63, *P* = 0.003). RMR was positively associated with FT4 in hyperthyroid state (*r* = 0.57, *P* = 0.01) but not in euthyroid state (*r* = 0.36, *P* = 0.12). FOX was positively associated with FT3 (*r* = 0.59, *P* = 0.01), FT4 (*r* = 0.52, *P* = 0.02) in hyperthyroid state and FT3 (*r* = 0.70, *P* = 0.001), FT4 (*r* = 0.52, *P* = 0.02) in euthyroid state (Table 4).

**Table 4:** Correlations between FT3 or FT4 and imaging parameters, RMR, and FOX at hyperthyroid and euthyroid state. *n* = 21, correlations were used to assess the relationship between variables after adjusting for weight.

	Hyperthyroid		Euthyroid	
	FT3	FT4	FT3	FT4
PET SUV mean (g/mL)	**0.58 (0.01)**	0.59 (0.01)	0.10 (0.67)	0.07 (0.78)
sFF (%)	0.32 (0.17)	−0.23 (0.33)	−0.02 (0.93)	0.14 (0.55)
Tscv mean (°C)	0.20 (0.39)	0.22 (0.35)	**0.55 (0.01)**	0.37 (0.11)
RMR (kcal/day)	**0.69 (0.001)**	**0.57 (0.01)**	**0.63 (0.003)**	0.36 (0.12)
FOX (g/min)	**0.59 (0.01)**	**0.52 (0.02**)	**0.70 (0.001)**	**0.52 (0.02)**

Significant correlations between variables are shown in bold with corresponding coefficient (r) and *P* values adjusted for body weight. Statistically significant values are in bold.

## Discussion

Hyperthyroidism is a hypermetabolic state leading to multiple changes in metabolism with increased resting energy expenditure (EE) and thermogenesis. BAT is involved in the regulation of EE. Thyroid hormones may have metabolic effects on BAT development and function. In our current study, we examined hyperthyroidism effects on BAT activity independent of cold stimulation. Our key finding was a small number of hyperthyroid patients had detectable BAT and that FT3 reduction was significantly correlated to BAT activity during the transition from hyperthyroidism to euthyroidism.

The effects of thyroid hormones on body composition have been described extensively in the literatures (33, 34). One of the main functions of thyroid hormone is the homeostasis of EE and BMR for the benefit of growth and development (35). The thyroid gland secretes thyroid hormones which increase BMR (36). Thyroid dysfunction is associated with changes in energy homeostasis. In our study, the resting EE decreased significantly when hyperthyroid participants became euthyroid. This was accompanied by significant increases in body weight, fat mass, and lean mass, with no change in percentage body fat as was consistently reported previously (37, 38). In patients with hyperthyroidism, prior to ATD therapy, lean body mass was decreased which suggested that patients with hyperthyroidism had weight loss predominantly due to a decrease in lean body mass despite increased food intake (33, 38). Our study participants had a significant increase in lean body mass by ~2 kg when they achieved euthyroidism (from 33.8 ± 1.6 to 35.7 ± 1.8 kg). The magnitude of increase in lean body mass was greater than a fat mass increase (from 19.5 ± 1.6 to 20.3 ± 1.7 kg) probably due to a decrease in thermogenesis and protein catabolism. However, Chng *et al.* (39) reported a significant increase in percentage body fat with no change in fat-free mass after achieving euthyroidism in Chinese women in Singapore. Perhaps the slightly different gender and ethnic proportions may have contributed to the inconsistency. Other metabolic parameters changes including glucose, insulin, and cholesterol levels in our study participants were consistent with the findings of the previous study (39).

^18^F-FDG PET has been regarded as the gold standard of imaging BAT. Magnetic resonance (MR) imaging and infrared thermography (IRT) have more recently emerged as safer alternatives for serial, quantitative measures of BAT. In our previous study (24), we have demonstrated that MR and/or IRT can serve as effective non-ionizing alternatives to ^18^F-FDG PET to evaluate BAT activity after cold stimulation. In our current study, we used ^18^F-FDG PET, MR FF, IRT Tscv, and whole-body calorimetry to detect BAT activity without cold stimulation. We found that only MR FF was significantly increased from hyperthyroid to euthyroid status which is consistent with a decrease of BAT activity in all the participants with the restoration of euthyroid levels of thyroid hormones. We found that 8 of 21 early stage hyperthyroid patients showed detectable ^18^F-FDG PET uptake which we defined as BAT-positive participants. The method using FDG/PET to measure BAT activity was rather diverse. Until more recently, the cut-off SUV value to define BAT-positivity is also varying in different research groups. In our previous study (32) and other studies (40, 41) in healthy participants after cold exposure, the cut-off value is SUV max of 2.0. However, in our current study, we found the thyroid hormones induced significantly less BAT activity compared with cold exposure in our previous study (32). Therefore, based on the BARCIST 1.0 (BAT criteria in Imaging studies) and other studies (29, 30), we adopted SUV max of 1.5 as the cut-off value to define BAT-positive and BAT-negative groups.

During the transition from hyperthyroid to euthyroid status, PET SUV max, and IRT Tscv did not change. It is well known that cold exposure is by far the most potent stimulus for BAT and following activation, an increase in EE can be measured (41). Upon cold exposure, ^18^F-FDG uptake and BAT temperature changes correlate well with BAT activity by PET and IRT methodologies as exploited for most BAT imaging (30) which is consistent with our previous report (24). Hence, ^18^F-FDG PET and IRT may be better suited to detect BAT activity post-cold exposure, unlike our present study which involved no cooling stimulus. MR FF has also been used to study BAT. Also, ^18^F-FDG is not a sensitive indicator of BAT activity compared with fatty acid-based radiopharmaceuticals such as 14(R,S)-[^18^F]-fluoro-6-thia-heptadecanoic acid (^18^FTHA), given that activated BAT oxidizes fatty acids as the predominant fuel rather than glucose (42). This is important since a selective impairment of glucose but not fatty acid uptake by BAT has been shown in a study on patients with diabetes (43). Hence, all earlier published human studies that did not reveal BAT activity with changes in thyroid status may lack the necessary PET-CT sensitivity for BAT detection due to the use of ^18^F-FDG instead of fatty acid tracers such as ^18^FTHA (43). Notably, MR FF does not require cold exposure to detect BAT activation (44). In our current study, we found that MR FF was increased in both BAT-positive and BAT-negative (as defined by FDG-PET) participants as they transitioned from the hyperthyroid to the euthyroid state, which suggested a reduction in BAT activity after antithyroid drug treatment. This was accompanied by a reduction of RMR and fat oxidation as shown by calorimetry, as well as an increase in fat mass, lean mass, and superficial s.c. adipose tissue depot volume (Table 1). Hence, MR FF may be a more sensitive method to study BAT activity without cold exposure compared with FDG PET and IRT. However, partial volume effects need to be taken into account when performing fat fraction measurements with MRI (45, 46). There is still no widely acceptable, optimized approach to study human BAT. More research is needed to explore different approaches to study BAT under different conditions.

Thyroid hormones, long recognized as crucial mediators of thermoregulation, play an important role in BAT development and function, the latter being a major site of facultative thermogenesis (47). Thyroid hormones and the sympathetic nervous system (SNS) act either independently or synergistically to change the availability of fuel substrates and the expression of UCP-1 in BAT in infants and small mammals (47). Masini *et al.* (48) earlier reported experimental hyperthyroidism-induced modification of BAT in rats which proved the critical function played by thyroid hormones for BAT growth and activity. Similar studies in humans are limited as iatrogenic induction of thyrotoxicosis for clinical research is often not ethically tenable in most countries. Minna *et al.* (20) reported hyperthyroid participants had higher BAT glucose uptake compared with healthy participants, which supported BAT activation by elevated thyroid hormones. Some adult human studies also demonstrated a higher level of thyroid hormone nuclear receptors expressed in activated BAT, implying a role for thyroid hormone in the regulation of BAT activation (49). Many previous studies relating the thyroid with BAT were conducted using cold stimulation (50, 51). Notably, Evie *et al.* (52) reported that BAT activity significantly increased in the subclinical hyperthyroid state compared to the hypothyroid state. Expectedly, it is difficult to examine thyroid hormone effects on BAT under normal euthyroid conditions.

Enigmatically, Zhang *et al.* (21) reported that they did not find active BAT in any hyperthyroid patients and postulated that abnormally high circulating thyroid hormone may not necessarily increase BAT activity. However, at least 38% of our hyperthyroid patients had detectable BAT activity. In our subjects, PET SUV max was significantly higher in BAT-positive participants compared to BAT-negative participants. Additionally, MR FF was significantly lower in BAT-positive participants compared to BAT-negative participants. IRT Tscv was also significantly higher in BAT-positive relative to BAT-negative participants. Our participants are newly diagnosed with Graves’ disease. However, the duration of hyperthyroidism of each participant was not clearly known and this may have contributed to the inconsistency. Regarding the lack of active BAT among the remaining 13 hyperthyroid patients, it is possible that ^18^F-FDG uptake at classical BAT depot sites might have diminished below the SUV threshold because these patients were recruited after antithyroid drugs had been initiated for 3–4 weeks. Santhanam *et al.* (53) reported in a systematic review that BAT detection by ^18^F-FDG PET might be dependent on ambient intracellular T3 levels within the adipose tissue. We detected an increase in the fat fraction by MR FF in sBAT during the transition from hyperthyroid to euthyroid status in every subject, thereby inferring a decrease in BAT activity as thyroid hormones are being normalized. The relationship between thyroid hormone and BAT in adult humans evidently needs further investigation. As a corollary to this, we established the existence of a BAT-thyroid axis and demonstrated the existence of a physiological feedforward loop in euthyroid people important for thermoregulation (42).

Some animal studies concluded that T3, besides inducing the known modifications in the lipid content of BAT, may induce a very early proliferation and differentiation of brown adipocyte precursors, like that observed during exposure to cold (48). High concentration of T3 is necessary for the recruitment of brown adipocytes and up-regulation of UCP1. BAT contains an abundant amount of type II 5’ iodothyronine deiodinase (DIO_2_) which converts T4 to active T3 (54). In our previous study, we had reported that FT3 level at baseline was significantly higher in BAT-positive healthy participants than BAT-negative participants (55). Junker *et al.* (56) reported that biologically active T3 may be involved in the development of supraclavicular BAT. In our current study, we also found that circulating FT3 concentration was significantly higher in BAT-positive patients compared to BAT-negative patients in both the hyperthyroid and euthyroid states. There was a significant interaction between the patients’ status and BAT status for FT3. As such, a reduction in FT3 expectedly leads to a lower BAT activity. FT3 concentration during a hyperthyroid state was positively associated with sBAT PET SUV mean. We have previously proposed that a higher thyroid hormone output by the thyroid results in greater brown adipocyte differentiation and BAT activation, which in turn increases the probability of higher thyroid hormones being found among those who are BAT-positive (55).

The present study has some limitations. First, cold exposure was not applied to the participants before the ^18^F-FDG PET/MRI and IRT. Hence, the BAT activity is lower than what we have anticipated. The comparable effects before and after treatment were not sufficiently disparate such that the level of detection of BAT via ^18^F-FDG PET and IRT was relatively limited compared to MRI. Secondly, MR FF is more sensitive in demonstrating BAT activity changes in this study of hyperthyroid patients. The MRI-based static FF is known to be significantly limited by partial volume effects, such as due to voxel size limitations. MR FF cannot differentiate between intracellular water content and non-lipid tissue portions (e.g. from vessels) within a voxel. Very small vessels, lymph nodes, and adjacent muscles are difficult to be excluded especially in the supraclavicular adipose tissue. Thirdly, as the patients were not recruited upon the first diagnosis of hyperthyroidism but within a brief 1–4 weeks of initiating carbimazole/thiamazole, the duration of thyroid hormone dysfunction for each participant was different depending on the time they were prescribed antithyroid drugs by their doctors. Hence the duration of hyperthyroidism before participating in our study was different and this may have contributed to some results falling below the level of statistical significance.

In summary, hyperthyroidism is often associated with activated BAT which is better demonstrated using MR FF and whole-body calorimetry compared to ^18^F-FDG PET or IRT without cold stimulation. With the attainment of euthyroidism, there is an accompanying reduction of BAT activity.

## Declaration of interest

The authors declare that there is no conflict of interest that could be perceived as prejudicing the impartiality of this study.

## Funding

The study was supported by the Singapore Ministry of Health http://dx.doi.org/10.13039/100009647’s National Medical Research Council http://dx.doi.org/10.13039/501100000265 (NMRC) Clinician Scientist Award (grant ID NMRC/CSA-INV/0003/2015) and the Agency for Science, Technology and Research http://dx.doi.org/10.13039/501100001348 (A*STAR) awarded to A/Prof. Melvin Leow.

## Author contribution statement

L J S conducted the study, analyzed the data, interpreted results, and wrote the manuscript; H J G and P G conducted the study; S K V, S A S, and N M analyzed the data; C J H and S S V designed the study, provided intellectual inputs and critically reviewed the manuscript; P S Y, Y S L, B S P L, A S, W H H, T P E Q, C K C, D E K C, and R D recruited the patients, edited and critically reviewed the final manuscript; M K S L conceived and designed the study, assisted in data interpretation, provided intellectual inputs, edited and critically reviewed the manuscript. All authors contributed to editing the final manuscript and approved the final manuscript for publication.
